# A Feasible One-Step Synthesis of Hierarchical Zeolite Beta with Uniform Nanocrystals via CTAB

**DOI:** 10.3390/ma11050651

**Published:** 2018-04-24

**Authors:** Weimin Zhang, Weixing Ming, Sufang Hu, Bo Qin, Jinghong Ma, Ruifeng Li

**Affiliations:** 1 College of Chemistry and Chemical Engineering, Taiyuan University of Technology, Taiyuan 030024, China; zhangweimin_1989@126.com (W.Z.); mingweixing@163.com (W.M.); husufang0114@link.tyut.edu.cn (S.H.); rfli@tyut.edu.cn (R.L.); 2 Dalian Research Institute of Petroleum & Petrochemicals, SINOPEC, Dalian 116045, China; qinbo.fshy@sinopec.com

**Keywords:** hierarchical zeolite, zeolite Beta, zeolitic nanocrystals, CTAB, hydrothermal synthesis, naphthalene benzylation

## Abstract

A hierarchical zeolite Beta has been prepared by a feasible one-pot and one-step method, which is suitable for application in industrial production. The synthesis is a simple hydrothermal process with low-cost raw materials, without adding alcohol or adding seeds, and without aging, recrystallization, and other complex steps. The hierarchical zeolite Beta is a uniform nanocrystal (20–50 nm) aggregation with high external surface area (300 m^2^/g) and mesoporous volume (0.50 cm^3^/g), with the mesoporous structure composed of intercrystal and intracrystal pores. As an acid catalyst in benzylation of naphthalene with benzyl chloride, the hierarchical zeolite Beta has shown high activity in the bulky molecule reaction due to its introduction of mesostructure.

## 1. Introduction

Zeolite Beta, with its three-dimensional 12-membered ring (12R) microporous structure/network, is one of the three large pore zeolites. Compared with the other two zeolites Y and Mordenite (MOR), zeolite Beta possesses a relatively open pore channel system (12 R × 12 R × 12 R), high Si/Al ratio and strong acidic sites, implying high thermal and hydrothermal stability [1]. In fact, as acidic materials, the zeolite has shown a lower capacity to promote reactions involving hydrogen transfer and coke production in vacuum gas oil cracking and hydrocracking, displaying great potential as solid acid catalyst in chemical and petrochemical processes. However, as with other zeolites, zeolite Beta has micropores typically smaller than 1 nm, which limits the diffusion of compounds within these pores and the size of molecules that can be catalyzed, for which the mass-transfer problem cannot be ignored when bulky molecules are involved in the reactions catalyzed by microporous zeolites [2]. In recent years, researchers have devoted great efforts to designing microporous-mesoporous or hierarchically structured zeolite to reduce the diffusion path of reactants and products in the zeolite phase to expand its applications [3].

Two main approaches have been employed to impart mesoporosity to zeolites [4]: (1) top-down techniques for removal of silica and/alumina from the framework of zeolites by desilication and/or dealumination; and (2) bottom-up procedures to utilize the organic soft or the inorganic hard templates. The first is a destructive approach that often results in loss of zeolitic micropores and acid sites. The second strategy is a convenient and versatile approach, in which the inorganic hard templates, e.g., various types of carbon materials such as carbon nanotubes, carbon nanofibers, ordered mesoporous carbons, etc. were usually used to create mesopores inside the bulk zeolites, though the organic soft templates were usually favored due to their compatibility and variety of the synthesis systems, and possibly practical applications in industry. In application, organic amphiphilic molecules or surfactants are frequently employed in the hydrothermal syntheses of porous zeolites for the construction of enhanced pore systems (mesopores or macropores) beyond the intrinsic micropores. The “soft-templating” method is widely used to generate hierarchical zeolites, which usually involves the use of relatively flexible organic molecules such as polymers and surfactants serving as the mesopore templates.

The synthesis of mesoporous zeolite beta materials has been approached through several of the methods mentioned above. Of these, the most conventional surfactant (cetyltrimethyl ammonium bromide, CTAB), a structural-directing agent for ordered mesoporous materials, such as MCM-41 materials, has been paid special attention frequently for constructing the hierarchical zeolite Beta with meso- and microporosity. Early in 1994, Beck et al. [5] explored the ability of alkyltrimethylammonium surfactants of the type CnH_2n+1_(CH_3_)_3_NBr which serve as the structure directing agents, or templates, for the formation of microporous or mesoporous molecular sieve frameworks and found that single-surfactant molecules serve to direct the formation of microporous materials whereas mesoporous molecular sieves, such as MCM-41, are formed by surfactant aggregates. The short-chain surfactant molecule (Cn < 8) appears to function more like a single-chain, pore-filling agent related to even smaller alkyl chain-length quaternaries such as tetramethylammonium, tetraethylammonium, etc. The resultant product is microporous. Here, micellar and liquid-crystal structure formation would be favored for the larger alkyl chain length surfactants, resulting in mesoporous products. 

Bagshaw et al. [6] developed a one-pot and two-step method for the synthesis of a composite material with highly crystalline zeolite Beta and highly ordered mesoporous material (MSU-SBEA), in which the zeolitic phase formation is primarily controlled by a pre-crystallization zeolite seed reaction step, while an alkalinity (KOH) reduction step is the key to the creation of highly ordered mesostructured phase via CTAB. Using colloidal precursor containing secondary building units of zeolite Beta as the silica and aluminum sources, small crystal zeolite Beta was synthesized in a biphasic H_2_O-CTAB-alcohol system [7]. A solution-mediated process was thus proposed, in which CTA^+^ ions were enriched at the liquid–solid interface through the strong electrostatic force between the polar head groups of CTA^+^ and the negative charged microcrystal surface, hindering the further growth of zeolitic microcrystals. In Serrano’s research group, hierarchical zeolite Beta was prepared by employing the reorganization of the protozeolitic nanounits by incorporation of a surfactant (cetyltrimethylammoniumbromide, CTAB). The formation of protozeolitic nanounits was prevented by pre-crystallization of the starting gel. The hierarchical zeolite Beta shows a narrower mesopore size distribution, more uniform and stable tetrahedral Al species and a high acid strength [8]. Very similar strategies of synthesis for the generation of mesopores, the pre-crystallization and the reorganization with the action of CTAB, were used to prepare other hierarchical zeolites, such ZSM-5 [9,10], TS-1 [11], Y [12,13], etc. Similarly, other organic molecules and surfactants, polydiallyldimethylammonium chloride (PDDA) [14], N_2_-p-N_2_ [15], etc. were employed to synthesize hierarchical zeolite Beta in aqueous and alcohol system.

In our laboratory, mesoporous zeolite Beta was hydrothermally directly prepared using silanizing silica without any mesoporus templates by the bond-blocking principle [16]. However, the high cost of the templates and rigorous procedures may be a concern for the large-scale applications and the commercial development of the hierarchical zeolites prepared by several of the methods mentioned above is in urgent need. In the present work, we report a facile synthesis method, one-pot and one-step, for the preparation of hierarchical zeolite Beta with different size distribution, in which only conventional silica and alumina sources, template and CTBA are used, with only a simple hydrothermal process applied in a sole aqueous system. Several key factors in the synthesis are investigated. The scale-up test in a 10 L autoclave for the preparation of the hierarchical zeolite Beta demonstrates the practicability in industrial use.

## 2. Materials and Methods

### 2.1. Materials

Silica (SiO_2_, Aerosil200, Degussa, Marl, Germany), aluminate sodium (Al_2_O_3_ 41%, Qingdao Haiyang Chem. Eng. Ltd., Qingdao, China), tetraethylammonium hydroxide (TEAOH, 20 wt. % solution in water), cetyltrimethylammonium bromide (98%) (CTAB), DTAB and NaOH (98 wt. %) were applied without further purification for the zeolite Beta synthesis.

### 2.2. Synthesis of the Mesostructured Zeolite

All zeolites were prepared using the hydrothermal method. The synthesis process is described as follows: sodium hydroxide and sodium aluminate were mixed with deionized water in a polypropylene flask and stirred for 5 min. Afterwards, tetraethylammonium hydroxide solution and cetyltrimethylammonium bromide were successively added to the previous solution and was stirred for 5 more minutes. Subsequently, silica was slowly added in under constant stirring. Then, the reaction mixture was stirred for 3 h at room temperature. Then, the resulting gel was introduced into a Teflon-lined stainless steel autoclave and treated hydrothermally at 140 °C for 10 d under static conditions. After the desired crystallization time, autoclaves were quenched, and the solid samples obtained was filtered and washed with deionized water until neutral pH was reached. CTAB and water contents in the synthesis gel were varied when using the reaction mixtures with the composition 2.6Al_2_O_3_:100SiO_2_:4Na_2_O:26TEAOH:(0.15–9.2)CTAB(DTAB):(930–2280)H_2_O. The wet filter cake was dried at 100 °C overnight. The calcination of the samples was carried out at 200 °C for 2 h (2 °C min^−1^) and at 550 °C for 8 h (5 °C min^−1^). The same synthetic technology and conditions were imitated in the scale-up test of the zeolite in a 10 L autoclave.

### 2.3. Preparation of the Catalysts

The calcined zeolites were converted to their acid form by ammonium ion-exchange in a 0.2 M NH_4_NO_3_ solution during 3 h at 50 °C (50 mL g^−1^ of zeolite). After three consecutive ion exchanges, the materials were washed, dried, and calcined at 550 °C for 5 h with a heating rate of 5 °C min^−1^, to obtain the active proton-form. Results of chemical analyses confirmed that most of the sodium cations were exchanged and its content reduced to less than 0.01 wt. %. The zeolites in their acid form were labeled as H-MZB-x (x represents the various changed synthetic parameter) as acid catalysts.

### 2.4. Characterization Methods

Zeolitic samples were characterized by powder X-ray diffraction (XRD) patterns on a Shimadzu XRD-6000 diffractometer equipped with Ni filter using CuKα radiation (λ = 0.15406 nm) with the step size and the counting time of 0.02° and 10 s, respectively. Nitrogen adsorption/desorption isotherms at −196 °C were obtained in a Quantachrome NOVA 1200e. Prior to the analysis, the samples were outgassed at 350 °C under vacuum for 5 h. The apparent surface area was obtained by the BET equation in the range between p/p_0_ = 0.02–0.15, whereas the external surface area and micropore volume were calculated by the t-plots in the range between 0.12 and 0.4 p/p_0_ (de Boer). The total pore volume was evaluated at p/p_0_ = 0.99. The mesopore volume (V_meso_) was calculated from the difference between the Vtot and Vmicro. Pore size calculations were performed with the DFT method on the adsorption branch using nitrogen on silica, cylindrical pore NLDFT model. Morphology analysis for zeolite was carried out using Field emission scanning electron microscope (SEM) images in a JEOL JSM-6700F (Tokyo, Japan). TEM images of the zeolite particles and catalysts were taken in a JEOL JEM-1011 (Tokyo, Japan). The samples were dispersed in ethanol and deposited on a holey carbon film supported on a Cu microgrid. Zeolite powder was immersed in ethanol and sonicated for about 30 min, with one to two drops spread on the copper grids covered with holey carbon.

### 2.5. Catalytic Tests

All the reactions were carried out in a mechanically agitated glass reactor of 50 mL total capacity a reflux condenser. The glass reactor (three-neck flask) was kept in an isothermal bath at the desired temperature and mechanically stirred at a given speed with an electrical motor. Standard reaction was carried out by dissolving 0.034 mmol naphthalene in cyclohexane of 30 mL as a solvent with 0.017 mmol benzyl chloride was added in to it. The reaction mixture was then transferred into the glass reactor fitted with a reflux condenser. The reaction was started at the set temperature after the addition of the 0.15 g catalyst. (A typical catalyst loading of 0.005 g/mL liquid phase volume was used). The H-zeolite catalyst was calcinated at 550 °C for 2 h before it was used. Analysis was performed on Gas Chromatograph (model HP-5) by using a 30 m × 0.38 mm × 0.5 μm) capillary column, coupled with a flame ionization detector.

## 3. Results and Discussion

**Figure 1:**
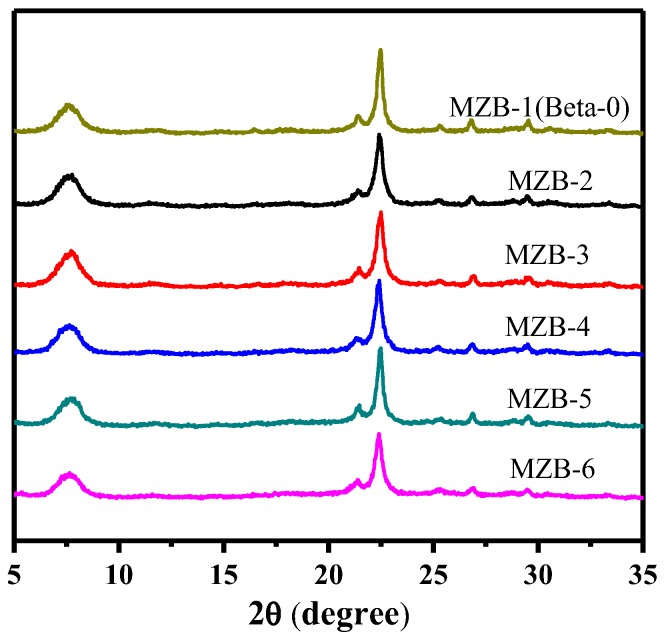
XRD patterns of as-synthesized zeolite Beta samples.

**Figure 2:**
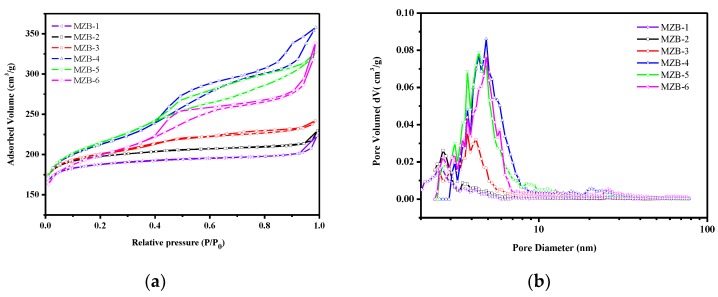
N2 adsorption-desorption isotherms of synthesized zeolite Beta samples (**a**) and pore distribution (**b**).

Conventional microporous zeolite Beta (MZB-1) was prepared according to the following chemical composition: 2.6Al_2_O_3_:100SiO_2_:4Na_2_O:26TEAOH:600H_2_O at 140 °C for 10 d under static conditions. To study the influence of the surfactant CTAB on the mesopore formation, different amount of CTAB were added in the crystallization system. Other Zeolite Betas (MZB-x, here x = 2–6) were synthesized by adding only CTAB in the star-mixture with the different CTAB/TEAOH ratios (see Table 1). The X-ray diffraction (XRD) patterns of the as-synthesized samples are presented in Figure 1. Diffraction patterns obtained for the samples are compared with the diffraction pattern reported in the database of zeolite structures. All synthetized zeolites have only the characteristic diffractions of zeolite Beta, with their main peaks at 2θ of 7.62°, 21.13° and 22.30°, showing pure Beta type crystalline phase to appear in those zeolitic samples, even if CTAB/TEAOH ratio changed in the rage of 3–36%. Textural characterization of the hierarchical zeolite Beta produced by surfactant CTAB were performed by using N_2_ physisorption experiments at-196 °C, as shown Figure 2. The parent zeolite Beta (MZB-1) producing a type I isotherm is a typical microporous zeolite. Other zeolite Betas obtained by the surfactant CTAB produce type I + IV isotherms, with the enhancive N_2_ uptakes at the relative pressure of approximately 0.4, indicating the presence of mesoporosity with the different mesopore-distribution. An important observation, the micropore volume diminishes slightly as mesoporosity develops, the mesopore volume raises by as much as 0.30 cm^3^/g, being 5 times more than the conventional microporous zeolite Beta (Table 1). The N_2_ uptake at relative pressures above 0.8 is caused due to the interparticle condensation in the large mesoporosity, presented in the isotherms of all the samples. When CTAB/THOH ratio changes from 12% to 24%, the external surface area reaches ca. 300 m^2^/g with high microporous surface area of 500 m^2^/g. These facts indicate that the formation of hierarchical pores is not achieved at the expense of the inherent microporous structure of zeolite Beta. These results validate the generation of mesoporosity from the intra-crystal of zeolites or the inter-crystal of zeolite aggregates assembled by small size of the zeolite particles (also see the following results of SEM and TEM).

**Figure 3:**
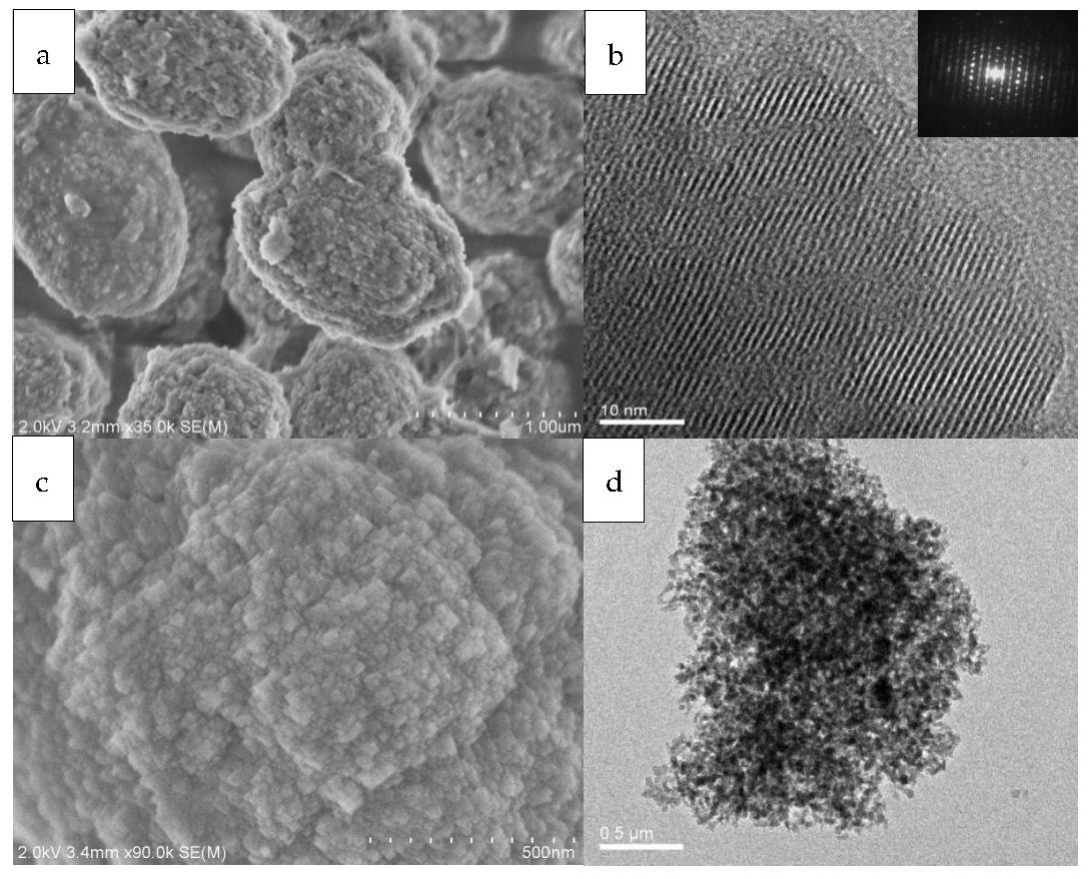
SEM and TEM images of synthesized zeolite Beta samples MZB-5. (**a**) dispersed particles(SEM); (**b**) particle structure (TEM); (**c**) particle surface (SEM); (**d**) particles aggregate (TEM).

The nanoscale morphology of the Beta zeolitic materials was elucidated by the scanning and transmission electron microscopy, as shown in Figure 3. In the range of the investigated CTAB/TEAOH ratios, the features of the samples are quasi-spherical with a diameter of about 1–2 μm and the surfaces appear rough, to be composed of zeolite beta nanoparticles imaged at higher magnification (Figure 3a,c). Individual particles in the aggregated spheres can be recognized with their sizes in the range of 50 nm (Figure 3). No amorphous SiO_2_ and/or isolated MCM-41 phases is found. Analysis with high-resolution transmission electron microscopy reveals that the particles are completely crystalline through the sample, as is substantiated by the lattice fringes with consistent orientations all over the specimen (Figure 3b), which were further identified by selected area electron diffraction (SAED) (inset of Figure 3b) that the particles are single crystals. The aggregates of the single-crystalline microporous nanoparticles then assemble into a well-crystallized mesoporous structure, which could be clearly observed from crystal morphologies. We have observed the existence of some intracrystalline mesopores in individual particles (Figure 3b) and intercrystalline mesopores in the aggregated sphere (Figure 3d). 

The effect of water content on the synthesis of the zeolite Beta materials in the hydrothermal system was investigated. XRD patters and adsorption isotherms of these samples were illustrated in respectively Figures S1 and S2. MZB-5 was used as the reference object, so it is feasible to reduce or increase the content of water in the system for preparing the hierarchical zeolite Beta. The results of the pore-parameters of the samples are listed in Table 2. The external surface area of the zeolite Beta increased with the increase of H_2_O content and without a marked reduction of microporous surface area. This means that the hierarchical pore-structure of the zeolite Beta can be adjusted by changing the water content or alkalinity of the synthesis system. Aggregates of about 50 nm crystallites resulted in a high mesopore volume (0.48 cm^3^/g) and a micropore volume (0.20 cm^3^/g) with a BET surface area of 816 m^2^/g. SEM images (Figure S3) have given the same results as the effect of CTAB, that is, the particles of the zeolite at the low magnification are relatively uniform in a nearly spherical shape with a diameter of about 1 μm; The micrometer-sized particles are an aggregate with the nanosized crystallites of a diameter of around 50 nm.

An opinion has been universally accepted that the pre-crystallization of the starting gel is necessary to induce the formation of protozeolitic nanounits for the assembly of the hierarchical zeolites in the CTAB-assisted system. In our tests, the effect of pre-crystallization time has been investigated according to the kinetic curves of the zeolite Beta crystallization in both the conventional and the CTAB-assisted systems. It is not hard to find that in the present synthesis system the pre-crystallization does not bring benefits for the hierarchical zeolite Beta (see Table 3). Therefore, the XRD pattern and SEM images have not been changed (Figures S4 and S5) in which the aggregated nanoparticles are about 50 nm in size, appearing in an elliptical outline shape.

The synthesis of hierarchical zeolite Beta in a H_2_O-CTAB-alcohol biphasic system were considered given that the nucleation through the formation of very small crystalline entities happened and crystal growth through the progressive incorporation of soluble species around these nuclei previously formed, and it can be concluded the surface CTAB-enriched microcrystals also aggregate through the weak dispersion force among the hydrophobic chains of CTAB [7]. With ethanol being a self-assembly modulator, a certain amount of ethanol hinders the overgrowth of zeolite crystals and slows down the crystallization process, which favors the self-assembly of zeolite subnanocrystals or nanocrystals under the direction by micro- and/or mesopore directing agents. We had added several alcohols such as ethanol, propanol or propanediol into the starting synthesis system and found that in our system alcohol is not a key factor for formation of the hierarchical zeolite Beta. The characterization results indicate that the hierarchical zeolite Beta in high quality can be obtained with or without alcohol (Table 4. XRD and N_2_ adsorption isotherms Figures S6 and S7 are supplied in the Supporting materials Section). In fact, the hydrophobic contribution of the alcohol additives plays an important role in the micellization of surfactant molecules, in which alcohols show an inhibitory effect on the formation of the micelles of CTAB. Micelles are formed by the self-aggregation of the surfactant molecules [17]. The surfactants can self-aggregate because of the presence of the distinct hydrophilic and hydrophobic parts within the molecule. Micellar solubilization is an important phenomenon and has attracted significant attention in the area of surface science and solution chemistry. However, alcohols considerably affect the critical micelle concentration (CMC) of CTAB and the degree of counterions bound to its micelles in their concentration range, beyond which they are present in the monomeric form in the solution [18]. That is the reason alcohol has not influenced the assembly of the hierarchical zeolite Betas in the CTAB-assisted system within our investigated range of alcohol/CTAB ratios.

Also, dodecayltrimethylaminiumbromide (DTAB) was used to replace CTAB in the synthesis system. Two typical recipes of MZB-4 and MZB-5 samples were employed in the synthesis system. The results are the samples MZB-12 and MZB-13 with DTAB as the surfactant-template shows very similar characteristics to those of the samples with CTAB. By contrast, the former has a higher external surface area accompanied by slightly depressed microporous surface area and pore-distribution (Table 5). Although CTAB micelles have a highly organized surface structure compared to the micelles of alkyltrimethylammonium bromides with shorter chain tails but less hydrated than DTAB. However, they are certainly present in monomeric form in the used solution.

Up to now, it is clear that hierarchical zeolite Beta can be prepared in a wide range by using a facial way, the one-pot and one-step method, without the pre-crystallization and the adding of alcohol in the synthesis system. The microporous template (TEOH) and the mesoporous template (CTAB or DTAB) simultaneously play a role in the reaction system for creating hierarchical zeolite Beta. The mechanism of zeolite nucleation has not been influenced by the increase of CTAB in the pure hydrothermal system. However, possibly because CTA^+^ competes with TEA^+^ for surface adsorption sites and occlusion into the precursor, there are decreases in the colloidal stability of the precursor nanoparticles and decreases in the nucleation rate (the role of ion pairs) [19].

To examine the feasibility and practicability of this strategy, an amplification test of the hierarchical zeolite Beta was accomplished in a 10 L autoclave under the same synthesis conditions. The characterization results of the amplified samples MZB-4 (M) and MZB-15 (M) show that the properties of the amplified products can match or overmatch those of the referents in the aqueous or alcohol-aqueous systems (Table S1). 

**Scheme 1:**
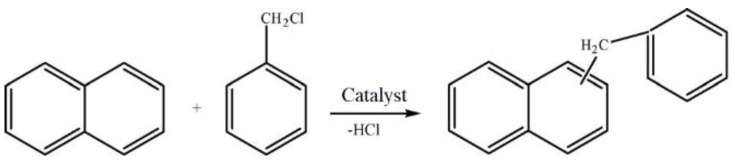
Benzylation of naphthalene with benzyl chloride over acid catalyst.

**Figure 4:**
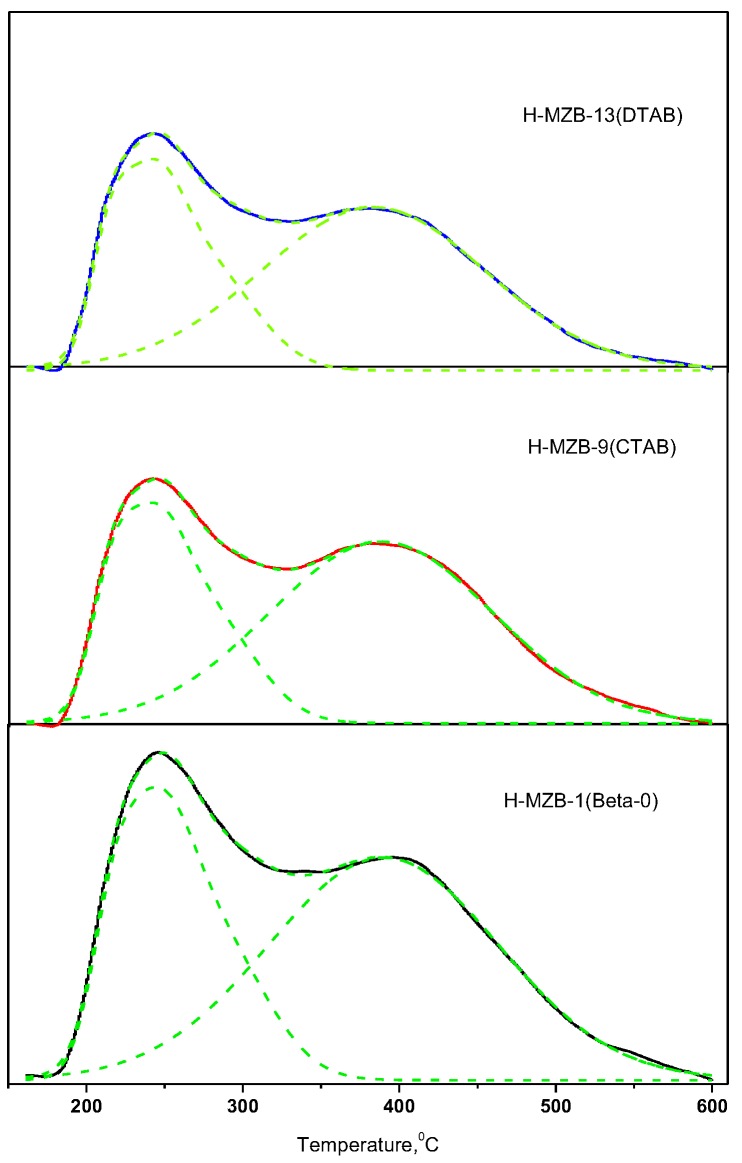
NH_3_-TPD curves of microzeolite Beta and hierarchical zeolite Betas.

**Figure 5:**
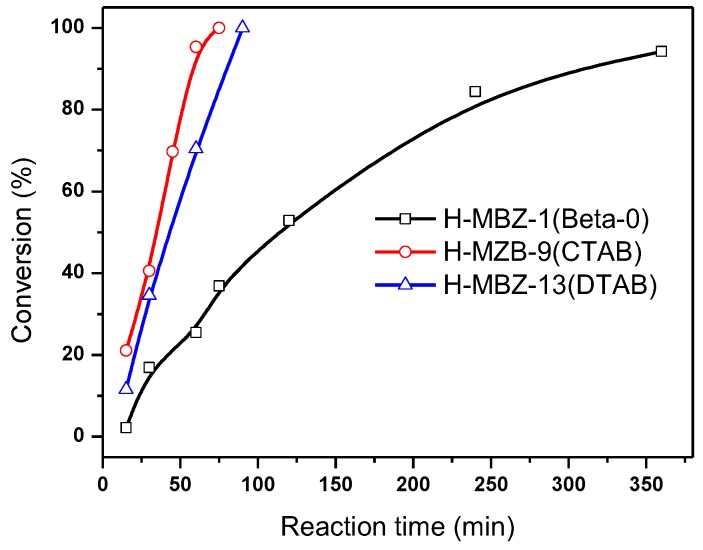
Benzylation of naphthalene with benzyl chloride over microzeolite Beta and hierarchical zeolite Betas.

The catalytic properties of the hierarchical zeolite Beta have been performed by a liquid phase benzylation of naphthalene with benzyl chloride (Scheme 1). The hierarchical zeolite Betas H-MZB-9 and H-MZB-13 were compared with conventional zeolite Beta H-MZB-1 (Beta-0) under the same reaction conditions. H-MZB-9 and H-MZB-13 are prepared by using CTAB and DTAB as mesostructured agents respectively and have very similar pore structure parameters and framework Si/Al ratios, 13.7 and 13.0, slightly less than 15.8 of MZB-1 (^29^Si MAS NMR spectra of the samples in Figure S8). The acidity of the H-MZB samples used in the reaction was determined by ammonia adsorption experiment. The NH_3_-TPD measurements of the samples are presented in Figure 4. The ammonia desorption curve outlines are very similar and indicate the presence of weak (T_max 1_, 240 °C) and strong (T_max 2_, 386 °C) acid sites in the samples. H-MZB-1 has higher total acid amount than H-MZB-9 and -13, but the three catalysts have very similar strong acid sites. Compared to H-MZB-1, H-MZB-9 and -13 have lesser weak acid sites. In benzylation of naphthalene with benzyl chloride, the hierarchical zeolite Betas showed much higher activity than that of the conventional zeolite Beta as listed in Figure 5. The catalytic activity of the hierarchical zeolite Beta is increased drastically because of the introduction of mesostructure. The conversion of benzyl chloride over the hierarchical zeolite Beta rises quickly with the reaction time and reaches 100% within 100 min. Using the conventional zeolite Beta, benzyl chloride cannot be completely converted even with a long reaction time of 360 min. Based on the acidity of the samples, the catalytic results are completely ascribed to the mesoporosity of the hierarchical zeolites. 

## 4. Conclusions

The hierarchical zeolite Beta with high external surface area can be synthesized in a hydrothermal system by a one-pot and one-step method via the assistance of CTAB, without aging and pre-crystallization, and without adding alcohol or using expensive raw materials. A key factor for the preparation is the reaction time due to the increase of CTAB and decrease of the nucleation rate. The amount of CTAB in the reaction mixture can change the ratio of the microporous and the mesoporous areas and the pore volumes. Pre-crystallization is not an important influence on the pore structure of the hierarchical zeolite Beta. The amount of H_2_O in the reaction mixture (pH value) has slight effect on the pore parameters of the hierarchical zeolite Beta. The addition of alcohol and substitution of DTAB for CTAB can obtain the hierarchical zeolite Beta with the same effect. Benzylation of naphthalene with benzyl chloride over the hierarchical zeolite H-Beta predicts the potential of application of the hierarchical zeolites in bulky molecule reactions.

## Figures and Tables

**Table 1:** Pore structure parameters of hierarchical Beta zeolites with different CTAB amounts.

Samples	CTAB/TEAOH (%)	S_BET_ (m^2^/g)	S_MIC_ (m^2^/g)	S_EXT_ (m^2^/g)	V_MIC_ (cm^3^/g)	V_MESO_ (cm^3^/g)	V_pore_ (cm^3^/g)	HF *
MZB-1	0	742	682	62	0.26	0.06	0.33	0.08
MZB-3	3	780	630	150	0.24	0.12	0.36	0.13
MZB-4	12	799	519	280	0.21	0.33	0.54	0.13
MZB-5	24	800	482	318	0.19	0.30	0.49	0.15
MZB-6	36	748	498	250	0.20	0.31	0.50	0.13

* HF is the hierarchical factor, defined as (V_mic_/V_total_) × (S_Ext_/S_BET_) [16].

**Table 2:** Pore structure parameters of hierarchical Beta zeolites with different H2O amounts.

Samples	H_2_O/Na_2_O	S_BET_ (m^2^/g)	S_MIC_ (m^2^/g)	S_EXT_ (m^2^/g)	V_MIC_ (cm^3^/g)	V_MESO_ (cm^3^/g)	V_pore_ (cm^3^/g)	HF
MZB-7	240	742	546	196	0.21	0.28	0.50	0.11
MZB-5	400	800	482	318	0.19	0.30	0.49	0.15
MZB-8	500	816	490	327	0.20	0.48	0.68	0.12
MZB-9	600	819	463	356	0.19	0.44	0.74	0.13

**Table 3:** Pore structure parameters of hierarchical Beta zeolites with different recrystallization time.

Samples	F-Time (h) *	S_BET_ (m^2^/g)	S_MIC_ (m^2^/g)	S_EXT_ (m^2^/g)	V_MIC_ (cm^3^/g)	V_MESO_ (cm^3^/g)	V_pore_ (cm^3^/g)	HF
MZB-5	0	800	482	318	0.19	0.30	0.49	0.15
MZB-10	30	803	544	258	0.21	0.31	0.52	0.13
MZB-11	80	743	593	149	0.23	0.14	0.38	0.12

* F-Time: First-step crystallization time at 140 C without CTAB or DTAB.

**Table 4:** Pore structure parameters of hierarchical Beta zeolites with and without alcohol.

Samples	S_BET_ (m^2^/g)	S_MIC_ (m^2^/g)	S_EXT_ (m^2^/g)	V_MIC_ (cm^3^/g)	V_MESO_ (cm^3^/g)	V_pore_ (cm^3^/g)	HF
MZB-14 (with)	763	530	233	0.21	0.27	0.48	0.13
MZB-4	799	519	280	0.21	0.33	0.54	0.13
MZB-15 (with)	739	374	365	0.15	0.38	0.54	0.14
MZB-9	819	463	356	0.19	0.44	0.74	0.13

**Table 5:** Pore structure parameters of hierarchical Beta zeolites with different TAB.

Samples	TAB/TEAOH (%)	S_BET_ (m^2^/g)	S_MIC_ (m^2^/g)	S_EXT_ (m^2^/g)	V_MIC_ (cm^3^/g)	V_MESO_ (cm^3^/g)	V_pore_ (cm^3^/g)	HF
MZB-1	0	742	682	62	0.26	0.06	0.33	0.08
MZB-12	12 (DTAB)	800	485	315	0.19	0.22	0.41	0.18
MZB-4	12 (CTAB)	799	519	280	0.21	0.33	0.54	0.13
MZB-13	24 (DTAB)	825	425	400	0.17	0.45	0.62	0.13
MZB-5	24 (CTAB)	800	482	318	0.19	0.30	0.49	0.15

## Supplementary Materials

The following are available online at http://www.mdpi.com/1996-1944/11/5/651/s1, Figure S1: XRD patterns of as-synthesized zeolite Beta samples with different H_2_O amount, Figure S2: N_2_ adsorption-desorption isotherms of synthesized zeolite Beta samples with different H_2_O amount (left) and pore distribution (right), Figure S3: SEM images of synthesized zeolite Beta samples with different H_2_O amount, Figure S4: XRD patterns of synthesized zeolite Beta samples with different pre-crystallization time, Figure S5: SEM images of synthesized zeolite Beta samples with different pre-crystallization time, Figure S6: XRD patterns of synthesized zeolite Beta samples with alcohol, Figure S7: N_2_ adsorption-desorption isotherms of synthesized zeolite Beta samples with alcohol (left) and pore distribution (right), Figure S8: Si MAS NMR spectra of microzeolite Beta MZB-1 and hierarchical zeolite Beta MZB-9, Table S1: Pore structure parameters of hierarchical Beta zeolites with 10L autoclave.
