# Supplementary material for: A Feasible One-Step Synthesis of Hierarchical Zeolite Beta with Uniform Nanocrystals via CTAB
Source: Materials (Basel). 2018 Apr 24;11(5):651. doi: 10.3390/ma11050651 (PMC5978028; doi:10.3390/ma11050651)
Supplement: Supplementary file 1 [file materials-11-00651-s001.pdf]

materials

Supplementary material:

A feasible One-Step Synthesis of Hierarchical Zeolite Beta with Uniform Nanocrystals via CTAB

Weimin Zhang [1], Weixing Ming[1], Sufang Hu[1], Bo Qin[2], Jinghong Ma [1,*] and Ruifeng Li[1]

1 College of Chemistry and Chemical Engineering, Taiyan University of Technology, Taiyuan 030024, China
2 Dalian Research Institute of Petroleum & Petrochemicals, SINOPEC. Dalian 116045, China
* Correspondence: majinghong@tyut.edu.cn; Tel.: +86-351-6111353

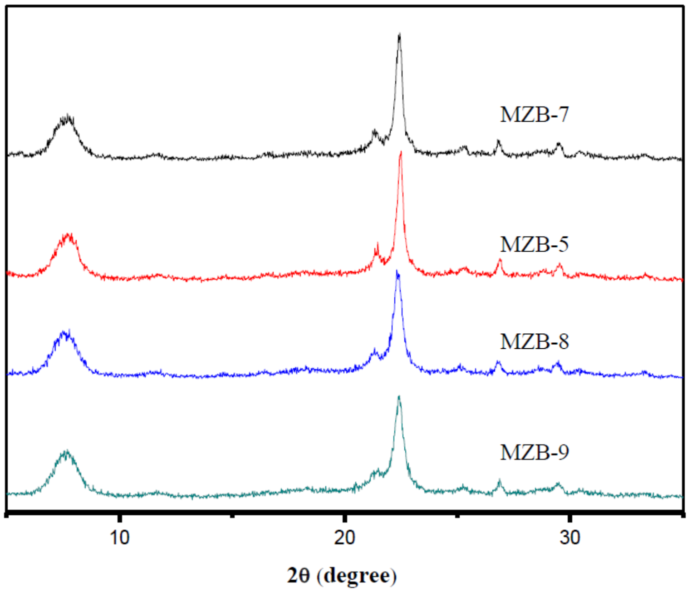

Figure S1. XRD patterns of as-synthesized zeolite Beta samples with different H_2O amount.

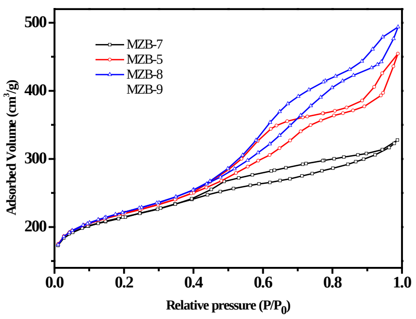

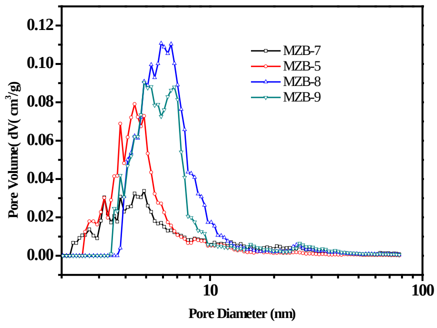

Figure S2. N_2 adsorption-desorption isotherms of synthesized zeolite Beta samples with different H2O amount (left) and pore distribution (right).

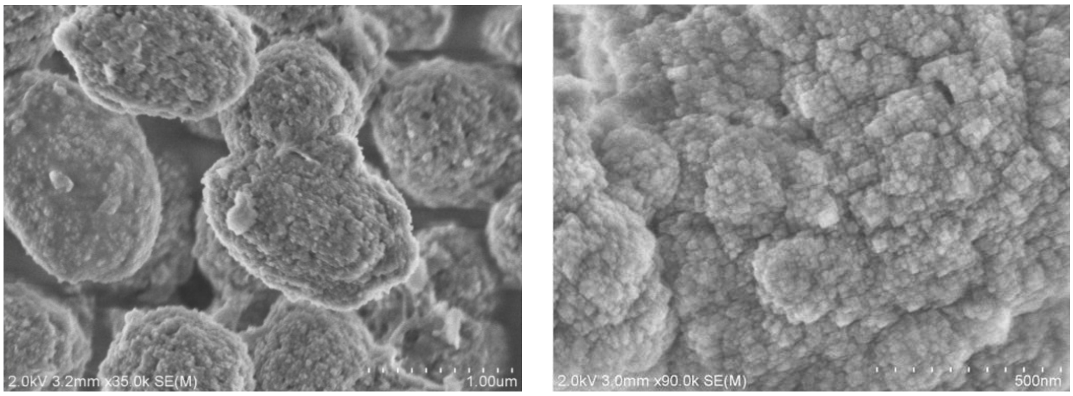

(**a**) (**b**)

Figure S3. SEM images of synthesized zeolite Beta samples MZB-5 (a) and MZB-9 (b) with different H_2O amount.

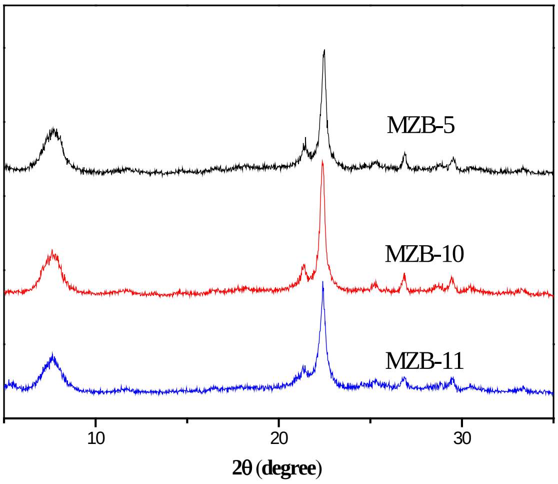

Figure S4. XRD patterns of synthesized zeolite Beta samples with different pre-crystallization time.

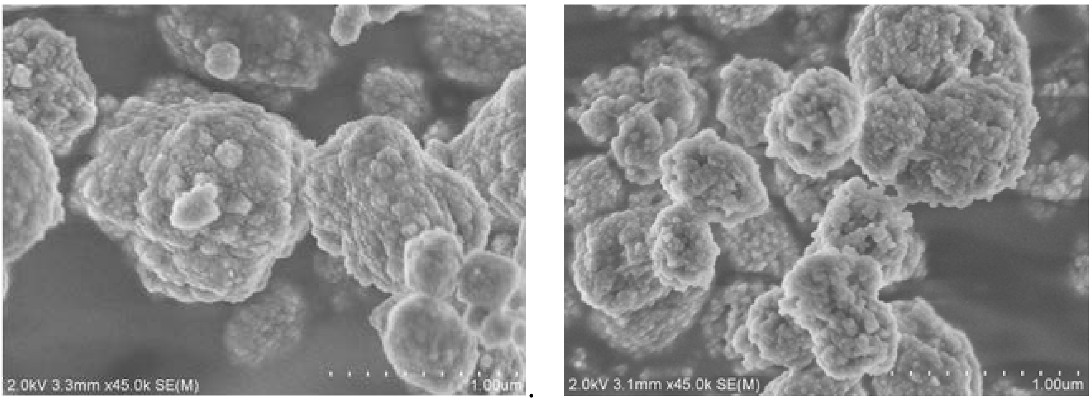

(**a**) (**b**)

Figure S5. SEM images of synthesized zeolite Beta samples MZB-10 (a) and MZB-11(b) with different pre-crystallization time.

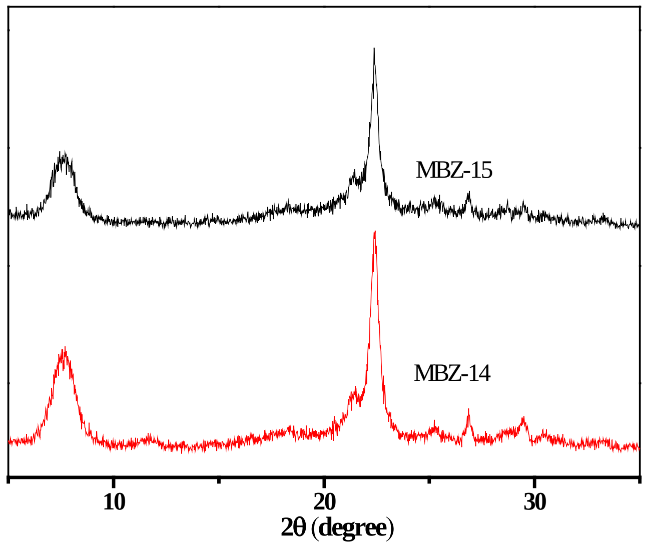

Figure S6. XRD patterns of synthesized zeolite Beta samples with alcohol.

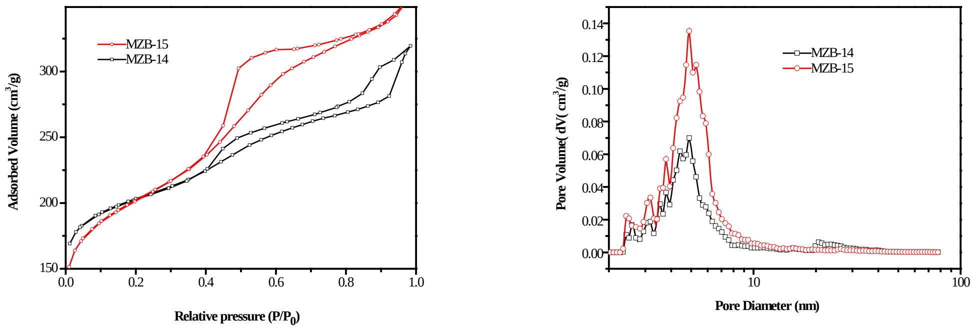

(a) (b)

Figure S7. N_2 adsorption-desorption isotherms of synthesized zeolite Beta samples with alcohol (left) and pore distribution (right).

Table S1 Pore structure parameters of hierarchical Beta zeolites with 10L autoclave.

Samples	S_{BET} (m^2/g)	S_{MIC} (m^2/g)	S_{EXT} (m^2/g)	V_{MIC} (cm^3/g)	V_{MESO} (cm^3/g)	V_{pore} (cm^3/g)	HF
**MZB-4 (M)**	**756**	**496**	**260**	**0.20**	**0.36**	**0.56**	**0.12**
**MZB-15(M)**	**715**	**394**	**321**	**0.16**	**0.53**	**0.69**	**0.10**

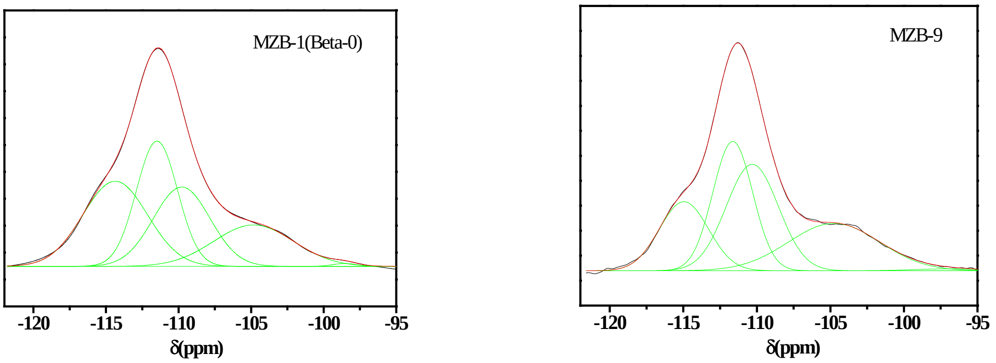

Figure S8. 29Si MAS NMR spectra of microzeolite Beta MZB-1 and hierarchical zeolite Beta MZB-9.
